# The Classroom Climate and the Role of Teachers’ Mentalized Affectivity: Mediation Through Job and Life Satisfaction

**DOI:** 10.3390/bs16081305

**Published:** 2026-08-01

**Authors:** Annalisa Levante, Flavia Lecciso, Ilaria Castelli

**Affiliations:** 1 Department of Human and Social Sciences, University of Salento, 73100 Lecce, Italy; flavia.lecciso@unisalento.it; 2 Lab of Applied Psychology, Department of Human and Social Sciences, University of Salento, 73100 Lecce, Italy; 3 Department of Human and Social Sciences, University of Bergamo, 24129 Bergamo, Italy; ilaria.castelli@unibg.it

**Keywords:** classroom climate, mentalizing ability, work satisfaction, life satisfaction, teacher, primary school, lower secondary education

## Abstract

The classroom climate is a multidimensional construct that embraces didactic practices, relational quality, shared norms, discipline, and a sense of belonging. A positive classroom climate conveys to students and teachers a feeling of safety and promotes a learning environment characterized by engagement, mutual respect, warmth, and a shared school vision. The current study, embracing the Prosocial Classroom Theoretical Model, aims to understand whether teachers’ socio-emotional competencies in terms of mentalized affectivity would affect their perception of the classroom climate via subjective dimensions of well-being in terms of life and job satisfaction. A total of 338 female teachers [*M* (*SD*) = 47.11 (10.17) years]—50.9% primary and 49.1% lower secondary education [*M* (*SD*)_years of experience_ = 17.79 (11.62)]—completed an e-survey. We tested a parallel mediation model in which teachers’ mentalized affectivity served as the potential personal resource for improving the classroom climate through the mediating role of job and life satisfaction. School grade and teaching experience were used as covariates. Results highlighted significant total, direct, and indirect effects. Although the cross-sectional design of the study limits causal inference, the preliminary findings are encouraging and may contribute to a better understanding of the resources that support the development of a positive school climate, with potential cascading effects on students’ socio-emotional and academic outcomes.

## 1. Introduction

The classroom climate is a multidimensional construct that embraces didactic practices, relational quality, shared norms, discipline, and a sense of belonging (Cohen et al., 2009; Grazia & Molinari, 2021). When the classroom climate is positive, students and teachers experience a sense of safety on social, emotional, and physical levels, encouraging their attendance in the school environment (Cheon et al., 2022). In contrast, a negative and hostile classroom climate leads to the potential for teachers and students to drop out due to the perception of an unsafe and boring place (Muñoz-Troncoso & Riquelme, 2025). Additionally, a positive classroom climate promotes a learning environment characterized by engagement, mutual respect, warmth, and a shared school vision among students, teachers, and families (Cohen et al., 2009).

According to Pianta’s (2001) model, the classroom climate results from the interaction of different systems from an individual level (i.e., the teacher’s or student’s characteristics) to a relationship level and up to a wider structural level (i.e., school policies). In the present study, we focus on the individual level and, specifically, on teachers’ key psychological aspects (i.e., mentalizing ability, job and life satisfaction) that may shape the classroom climate. The teaching profession carries a significant educational responsibility toward students’ growth (Kebritchi et al., 2017; Kingsford-Smith et al., 2023; la Velle, 2020; Macovei et al., 2023), and, at the same time, teachers face an increasing number of daily challenges (Levante et al., 2023; Cavioni et al., 2023; Nwoko et al., 2023; Safiye et al., 2023; Levante et al., 2025). Accordingly, their socio-emotional abilities, in terms of their mentalizing ability and well-being, and in terms of their perception of the quality of their life/job conditions, may play a pivotal role in how they efficiently cope with the requests and demands of positively managing a classroom. Consequently, the favorable classroom climate indirectly stimulates the teaching approach (DeWitt & Slade, 2014) and students’ achievement (Daily et al., 2019).

To elucidate the paths explored in the current study, the following section summarizes the empirical literature supporting the (1) direct relationships between teachers’ mentalizing ability, conceived as mentalized affectivity, and classroom climate, and the (2) potential influencing role of the teachers’ job/life satisfaction on this path.

In addition, the reasons supporting the focus on primary and lower secondary education teachers are discussed.

### 1.1. Mentalized Affectivity and Classroom Climate

The theoretical framework considered by the current study is the Prosocial Classroom model by Jennings and Greenberg (2009). The authors proposed a model in which the teachers’ socio-emotional competence and well-being directly and indirectly influence the classroom climate, the teacher–student relationship, socio-emotional learning, and the students’ academic and socio-emotional outcomes. In this complex theoretical model (for details see Jennings & Greenberg, 2009), the pivotal predictive role served by the teachers’ socio-emotional competence (as a personal resource) is crucial. The current study posited that mentalized affectivity is a potential teacher’s personal resource that affects the classroom climate.

Mentalized affectivity is a complex concept merging emotion regulation and mentalization abilities (D. M. Greenberg et al., 2017; Jurist, 2005, 2018), which are central to understanding and regulating emotions. Overall, emotional regulation is a wide-ranging term that describes explicit and implicit processes that involve monitoring, evaluating, altering, and modulating emotions (Gyurak et al., 2011) while being able to mentalize implies the human propensity in everyday social interactions to recognize that one’s own and others’ minds contain different mental states, and the ability to reason with those mental states to make sense of the behavior (Allen et al., 2008; Marchetti et al., 2013; Lecciso et al., 2016).

As a result, the teacher’s mentalized affectivity may be assumed as a personal resource supporting teachers to monitor their own emotions, fostering the students’ socio-emotional competencies (Bak et al., 2015; Bianco & Lecce, 2016), and managing the classroom climate effectively by also understanding classroom dynamics (Buyse et al., 2009; Hamm et al., 2011).

Nevertheless, it is worth noting that, to date, with respect to the classroom climate, only teachers’ mentalizing ability (Cavioni et al., 2023; Nwoko et al., 2023; Jennings & Greenberg, 2009; Cavioni et al., 2021) has been considered. For instance, the recent study by Rozenblatt-Perkal et al. (2025) reported that enhancing teachers’ mentalizing ability, due to a mentalizing-based group intervention, is a factor that affects emotional support as a component of the classroom climate. In contrast, to the best of our knowledge, no studies have explored the role of mentalized affectivity as a potential predictor of the classroom climate.

### 1.2. Job/Life Satisfaction and Classroom Climate

Looking at the Prosocial Classroom Theoretical Model (Jennings & Greenberg, 2009), not only teachers’ social and emotional competence, conceived in the current study in terms of mentalized affectivity, but also teachers’ subjective well-being is viewed as a teacher’s subjective dimension influencing the classroom climate. Overall, well-being is a multidimensional umbrella construct encompassing both hedonic and eudaimonic components of positive human functioning (Ryan & Deci, 2001). The hedonic dimension (i.e., subjective well-being) reflects the presence of positive affect, the relative absence of negative affect, and satisfaction with one’s life (Diener, 1984; Diener et al., 2018). Conversely, the eudaimonic dimension emphasizes psychological functioning, personal growth, and engagement in meaningful activity (Ryff, 1989). Although empirically distinguishable, these two facets are strongly interrelated and are best understood as complementary components of a single, broader construct (Huta & Waterman, 2014).

Therefore, from this complex conceptualization, the current study posited that job and life satisfaction are subjective dimensions of well-being potentially influencing the classroom climate.

Briefly, job and life satisfaction consist of feelings, beliefs, and behaviors toward one’s job (Weiss, 2002; J. Greenberg & Baron, 2000) and life (Diener et al., 2018), respectively.

Having a positive attitude toward their job leads teachers to perform it more effectively and enthusiastically (Demirel, 2014), and as a result, a high-quality education is produced. Therefore, job satisfaction may be a potential subjective dimension positively affecting the classroom climate. Regarding the concept of life satisfaction, to the best of our knowledge, no studies have explored the relationship with the classroom climate. In addition, a potentially strong reciprocal relationship between job and life satisfaction has been assumed (Demirel, 2014): job satisfaction affects life satisfaction, and increasing satisfaction gained from their job contributes to the general life satisfaction of teachers.

### 1.3. Mentalized Affectivity, Job/Life Satisfaction, and Classroom Climate

Evidence demonstrated the protective role served by mentalization ability for teachers’ overall well-being (Safiye et al., 2023; Schwarzer et al., 2023), and there is preliminary evidence that mentalizing is specifically linked to both life and job satisfaction (Levante et al., 2023; Cieślak et al., 2022). Nevertheless, to date, no studies have explored the role of job and life satisfaction as a subjective dimension of well-being in mediating the relationship between teachers’ mentalized affectivity and the classroom climate.

### 1.4. Teacher-Level Factors Affecting the Classroom Climate

Among teachers-level factors that may affect the classroom climate (e.g., the number of students in the class, the type of employment contract, part-time versus full-time, the number of class hours per week), the current study focused on the role played by the teachers’ age, teaching experience, and school grade in the classroom climate.

The relationship between teachers’ age and classroom climate remains a relatively underexplored dimension of educational research, often overshadowed by the more commonly studied construct of teaching experience, with which age is frequently confounded. Overall, taken together, the evidence suggests that teachers’ age influences the classroom climate largely indirectly (e.g., through its association with burnout risk and self-regulatory capacity) rather than through age itself, being a direct determinant of relational quality in the classroom. Recent findings (Pedditzi et al., 2021; Alghamdi & Sideridis, 2024) suggest that teachers’ age, associated with the cumulative emotional demands of the profession, may erode the personal resources needed to sustain a warm, supportive classroom climate. At the same time, age cannot be equated with a simple linear decline in teaching quality. Research using classroom observation tools found no evidence that novice teachers (i.e., 0–3 years of experience) were less competent than more experienced colleagues, although some decline in teaching quality emerged among teachers with four to five years of experience (Graham et al., 2020). This nuance is important for the classroom climate: younger and early-career teachers are not inherently disadvantaged in creating a positive classroom environment, and the trajectory of teacher effectiveness across the career span is non-linear rather than strictly age-dependent.

On teachers’ years of teaching, evidence suggests that it is widely regarded as one of the most influential teacher-level variables affecting the classroom climate, primarily through its association with classroom management skills, emotional regulation, and instructional quality. It is worth noting that, unlike age, experience captures the accumulation of practical, situated knowledge that teachers develop through repeated exposure to real classroom demands. Novice teachers appear to face specific difficulties in this regard. A recent observational study found that these teachers predominantly rely on reactive, teacher-directed strategies rather than proactive, student-centered approaches when managing disruptive behavior, and that this pattern harms both the teaching process and classroom climate (Karasova & Nehyba, 2025). The study by Koth et al. (2008) reported that experienced teachers are likely to feel more supported and more effective in their job compared to their younger counterparts. On the other hand, the study by Rozenblatt-Perkal et al. (2025) found no association between the teachers’ years of experience and emotional support as a component of the classroom climate. This inconsistency calls for further investigation.

Regarding the school grade, the evidence collected so far remains limited, weak, and often inconsistent (Nwoko et al., 2023). Nonetheless, teaching in primary or lower secondary education poses distinct challenges, given the complex and varied nature of teacher–student interactions (Collie et al., 2015). Indeed, during early (2–7 years) and middle (7–14 years) childhood (Brown & Beran, 2007), teachers act as extra-familiar caregivers, supporting not only pupils’ academic achievement (Wenglinsky, 2002), but also their emotional and social development (Kunter et al., 2013). Consequently, the demands and burden of supporting students’ development may negatively affect teachers’ performance and, in turn, classroom outcomes.

In summary, considering the short- and long-term consequences of the lack of teacher support for pupils (Pietarinen, 2000), and understanding what teacher-level factors need to be considered for teachers’ personal resources (e.g., mentalized affectivity) and subjective dimensions of well-being (e.g., job and life satisfaction), is a pivotal educational issue. Indeed, the chain effect may be damaging: the limited personal resources or subjective dimension of well-being perceived by the teachers may be closely related to a negative classroom climate, which indirectly affects teacher and student engagement, leading to teacher burnout (Levante et al., 2025; Carroll et al., 2022) and student dropout (Muñoz-Troncoso & Riquelme, 2025), respectively.

## 2. Materials and Methods

### 2.1. Aims and Research Questions

The novelty of the current study lies in conceiving the mentalized affectivity of teachers as a potential personal resource for the classroom climate, mediated through teachers’ subjective dimensions in the form of job and life satisfaction. The parallel mediation model was controlled by the significant teacher-level features as covariates. Considering the novelty of the study and the lack of similar evidence, two explorative research questions were formulated:

**RQ1**: Would the teacher’s mentalized affectivity be a potential personal resource for the classroom climate?

**RQ2**: Would the teacher’s mentalized affectivity be a potential personal resource of the classroom climate via the parallel mediation of job and life satisfaction?

**Figure 1:**
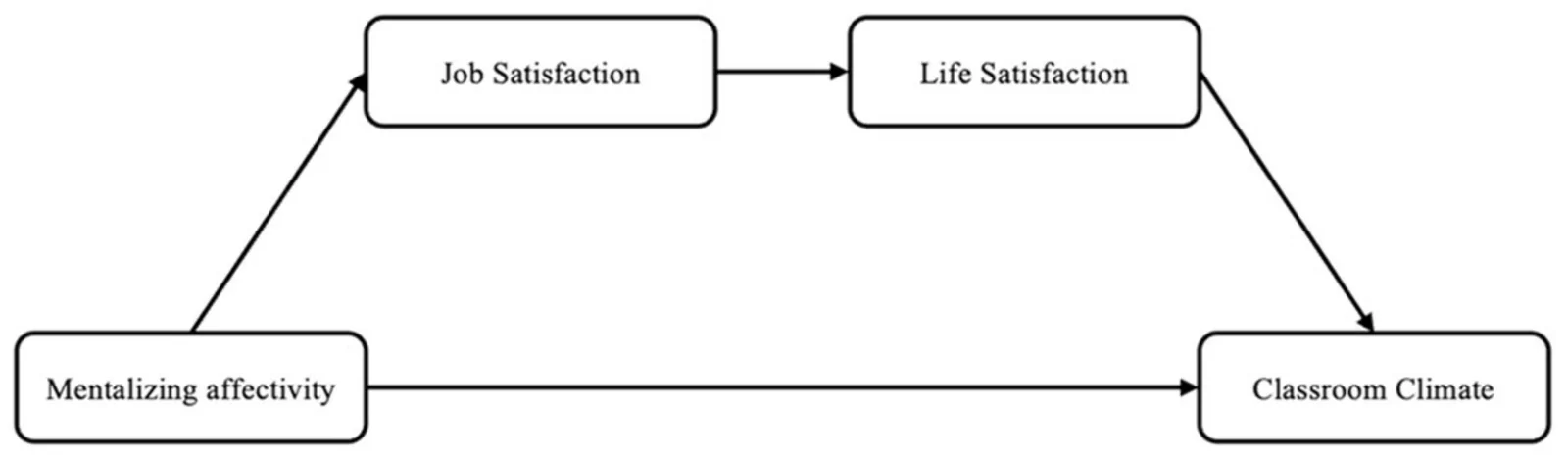
Hypothesized mediation model.

Figure 1 shows the hypothesized mediation model.

In addition, considering the potential role served by the teacher-level factors, according to the preliminary analyses, the teachers’ age, their length of teaching experience, and the school grade were explored as covariates in the parallel mediation model.

Considering the explorative nature of the study, descriptive analyses (i.e., group comparisons and associations between the teachers’ age, length of teaching experience, and the key variables) were also computed.

### 2.2. Procedure

The cross-sectional e-survey was filled in between September 2021 and January 2022 in provinces in the North and South of Italy. The e-survey was imported into Microsoft Forms, and the link was shared via mainstream social platforms (e.g., WhatsApp, Facebook) and an internal communication channel provided by the local school offices. We are aware of the potential flaws related to convenience sampling (e.g., scarce generalizability and selection bias); nevertheless, given the restrictions due to the COVID-19 pandemic at that time, it was mandatory.

The research team emailed the headmasters of local primary and lower secondary schools, requesting their agreement to disseminate the link among the teachers. To monitor the completion of the e-survey, a reminder was sent to the headmasters periodically. Participation was voluntary, and the questionnaire was filled out anonymously. The selection criteria used for recruiting samples were: (a) being a female teacher; (b) being employed in primary or lower secondary education; (c) being fluent in Italian. No age limit was applied. The first criterion was employed as, in Italy, teachers are mostly women: 95.6% and 77% in primary and lower secondary education, respectively (https://ec.europa.eu/eurostat/databrowser/view/educ_uoe_perd03/default/table?lang=en, accessed on 20 November 2022).

The study was conducted in compliance with the Italian rules and regulations for research in psychology, with the ethical requirements for research in psychology, and according to the GDPR (General Data Protection Regulation) and with the Code of Ethics of the World Medical Association (Declaration of Helsinki) for experiments involving humans. The Ethical Committee of the University of Salento and the University of Bergamo gave their approval (no. 71084/2021 and report n. 11 of 3 June 2021, respectively). Before answering the questions, participants gave their voluntary informed consent. Participants were also informed of the general research aims and their rights as participants. They knew that they could withdraw from the study at any time. No compensation was given for their participation.

### 2.3. Participants

A total of 390 e-surveys were completed by teachers (response rate); nevertheless, 37 teachers did not meet the inclusion criteria; that is, being employed in primary or lower secondary education schools. In addition, 15 of them did not sign the e-consent. A total of 338 questionnaires (completion rate) were collected from Italian female teachers [*M* (*SD*) = 47.11 (10.17) years]. The sample is balanced regarding the school grade: 172 (50.9%) of them were teachers at primary schools, and 166 (49.1%) were teachers in lower secondary education. Their educational level was clustered in accordance with the International Standard Classification of Education (ISCED). They reported an intermediate (ISCED Level 3—upper secondary education) level for 92 teachers and a high level (ISCED Level 6 and 7—bachelor’s or master’s level) for 182 teachers. Fifty-nine teachers reached a post-lauream title (ISCED Level 8—doctoral level). Regarding their marital status, 236 teachers had a partner, and 229 teachers had at least one child. Finally, the average length of their teaching experience was 17.79 years (*SD* = 11.62 years; range = 1–41 years).

### 2.4. Measures

#### 2.4.1. Mentalized Affectivity

To measure teachers’ mentalized affectivity, we adopted the Italian version of the Mentalized Affectivity Scale (D. M. Greenberg et al., 2017) devised by Rinaldi and colleagues (2021). This instrument is composed of 35 items, and the response options vary on a 7-point Likert scale from 1 (“completely disagree”) to 7 (“completely agree). This results in five scales, which form the current study’s internal consistency indices: (1) perception of the ability to identify (ω = 0.854; r = 0.324) their own and others’ emotions (example item “I am good at distinguishing between different emotions that I feel”); (2) tendency to express and communicate emotions (ω = 0.869; r = 0.399) with others (example item “If I feel something, I will convey it to others”); (3) interest/curiosity in recognizing and labeling emotions (ω = 0.845; r = 0.397) (example item “I am interested in learning about why I feel certain emotions more frequently than others”); (4) capacity to cognitively control and process emotions (ω = 0.829; r = 0.366) (example item “If a feeling makes me feel uncomfortable, I can easily get rid of it”); and (5) ability to remember personal childhood emotional experiences (ω = 0.769; r = 0.431) (example item “I can pinpoint childhood experiences that influence the way that I often think and feel”). A total score was computed as the mean of all items (ω = 0.873; r = 0.306). Evidence from the Italian validation (Rinaldi et al., 2021) showed that the measure has good internal consistency. All Cronbach’s alphas were adequate: “Identifying Emotions” = 0.86, “Expressing Emotions” = 0.84, “Curiosity about Emotions” 0.82, “Processing Emotions” = 0.79, “Autobiographical Memory” = 0.75. The fit of the CFA model was acceptable [(χ2 (584) = 1076.00, *p* < 0.001; RMSEA = 0.058; SRMR = 0.074)].

#### 2.4.2. Classroom Climate

To measure teachers’ perceptions of the classroom climate, the Italian version of the Multidimensional School Climate—Teacher Version by Grazia and Molinari (2021) was administered. The instrument is composed of 22 items, and response options varied on a 6-point Likert scale from 1 (“completely agree”) to 6 (“completely disagree). This results in five scales, which form the current study’s internal consistency indices: (1) the perception of the student relationship (*ω* = 0.917; *r* = 0.495) (example item “Students help each other”); (2) the student–teacher relationship (ω = 0.938; r = 0.640) (example item “In general, students and teachers get along well with each other”); (3) the educational climate (ω = 0.844; r = 0.431) (example item “Students are expected to give their best”); (4) a sense of belonging (ω = 0.85; r = 0.688) (example item “I feel comfortable”); and (5) interpersonal justice (ω = 0.705; r = 0.284) (example item “The rules are fair”). A total score was computed as the mean of all items (ω = 0.923; r = 0.592). Evidence of the Italian validation (Grazia & Molinari, 2021) showed that the fit of the CFA model is excellent [χ2(1259) = 2507.56, *p* < 0.001, RMSEA = 0.030, 90% CI [0.029, 0.032], CFI = 0.92, and SRMR = 0.05].

#### 2.4.3. Life and Job Satisfaction

For job and life satisfaction, two single-item measures were used for the study. They are “How satisfied are you with your life?” and “How satisfied are you with your job?”, with response options ranging from 1 (“not at all”) to 10 (“very much”). The single-item measures were previously administered successfully (De Carlo et al., 2024), supporting their ability to adequately assess the constructs of job and life satisfaction. To assess the potential presence of common method bias, given that the study variables were collected via self-report at a single time point, Harman’s single-factor test was conducted (Podsakoff et al., 2003). The single-factor test accounted for 15.4% of the total variance, well below the recommended 50% threshold, suggesting that common method bias is unlikely to represent a substantial concern in the present dataset.

### 2.5. Statistical Plan

The statistical analyses were performed using SPSS version 25. The power analysis to ensure the adequacy of the sample size was computed using the pamlj package 2.4.14 version for group comparisons and mediations. The sample size calculation using a priori power analysis based on conventional small-to-moderate effects of a medium effect size of 0.50, a desirable power of 0.90, and an alpha level of 0.050 (Bortolami et al., 2017; McClave & Sander, 2018) as a precaution was appropriate. Results indicated that a minimum of 86 observations are required to achieve a medium effect size of 0.50, a desirable power of 0.90, and an alpha level of 0.050 for group comparisons. In addition, a minimum of 137 observations are required to achieve a large effect size of 0.09, a desirable power of 0.90, and an alpha of 0.05 for the mediation model. Using conventional thresholds due to the lack of similar studies, the study sample size is adequate.

Regarding the Gaussianity of data distribution, Shapiro–Wilk tests showed that the data were non-normally distributed (mentalized affectivity: W = 0.358; classroom climate: W = 0.803; job satisfaction: W = 0.792; life satisfaction: W = 0.766). Therefore, the U-tests were computed for group comparisons, and Spearman’s rho was calculated separately for correlational analyses for teachers of primary and lower secondary education. To determine the potential predictive role of mentalized affectivity (X) on the classroom climate (Y) via the mediation of job and life satisfaction, parallel mediation models were performed using the PROCESS package for SPSS v3.0, applying Model 6. Due to the non-significant association between teachers’ age and study variables, the school grade and the length of teaching experience were included as covariates only. Given the non-normal distribution of the data, the mediation analyses were conducted using a bootstrapping approach. Specifically, indirect effects were estimated through bias-corrected bootstrap confidence intervals based on 5000 resamples (Hayes, 2017). Only standardized coefficients were reported. Because the format of the answer required participants to select an option to continue with the e-survey, no missing data were detected.

## 3. Results

### 3.1. Group Comparisons

Table 1 shows the mean scores, standard deviations, and statistical coefficients. Findings showed that teachers at primary schools reported significantly higher scores for the school climate compared to teachers working in lower secondary education. In other words, teachers working in primary schools reported better relationships with students, higher-quality relationships with students, and a better overall school climate. According to the results, the school grade was tested in the mediation model as a covariate.

### 3.2. Associations Between Study Variables in Teachers of Primary and Lower Secondary Education

Correlation analyses considering the length of teachers’ age, teaching experience, mentalized affectivity, classroom climate, and job and life satisfaction were computed for teachers of primary and lower secondary education separately (Table 2). In both subsamples, the mentalized affectivity shown by teachers was positively associated with their perception of the classroom climate and their levels of job and life satisfaction. In addition, the length of teaching experience was negatively associated with mentalized affectivity, job satisfaction, and life satisfaction in the subsample of primary school teachers. In contrast, the length of teaching experience was positively correlated with the classroom climate only in the subsample of teachers in lower secondary education. Lastly, teachers’ age did not correlate with any of the study variables. According to the results, only teachers’ length of experience was tested in the mediation model as a covariate, while teachers’ age was not included.

### 3.3. Parallel Mediation Model

**Figure 2:**
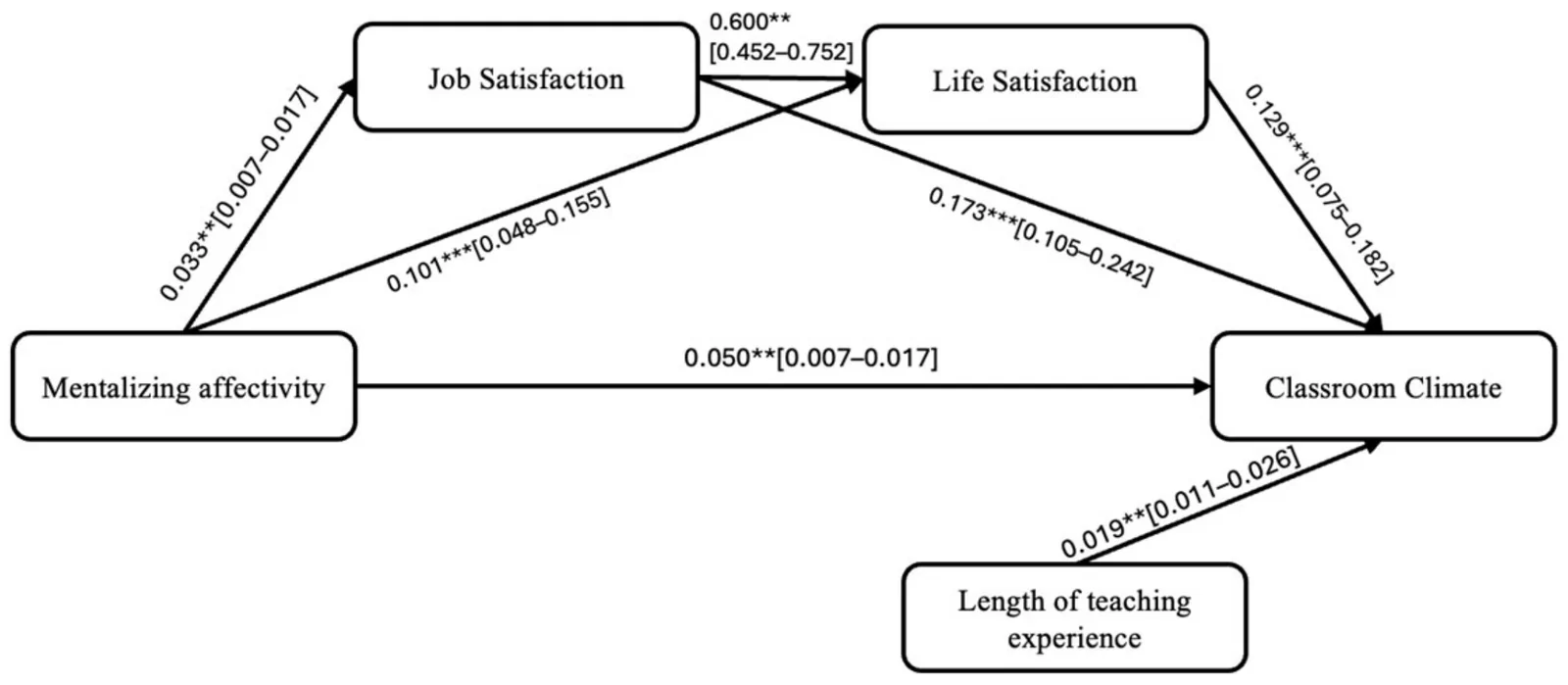
Results of the parallel mediation model. In brackets, the bootstrap confidence intervals are reported. Note ** *p* < 0.01; *** *p* < 0.001.

A mediation model was computed on the relationship between teachers’ mentalized affectivity ability and the school climate via the parallel mediation of job and life satisfaction. Figure 2 shows the results of the hypothesized model.

The parallel mediation model shown in Figure 2 is significant [F_(3,309)_ = 41.805; *p* < 0.001; R = 0.537; R^2^ = 0.289]. Table 3 reports all effect coefficients.

In other words, the findings revealed that the teachers’ mentalized affectivity positively impacted the classroom climate directly and indirectly, with job and life satisfaction acting as mediators. More precisely, the higher the teacher’s ability to interpret their own and others’ behaviors referring to mental states—in the form of mentalized affectivity—the more positive the classroom climate, because of the potential protective role played by two subjective dimensions, namely higher levels of job and life satisfaction.

Among the covariates included in the parallel mediation model, i.e., teaching experience and school grade, only the length of teaching experience positively impacts the classroom climate. This means that experienced teachers tend to report a more positive classroom climate.

## 4. Discussion

The vulnerability of the teacher population is well-documented (Gray et al., 2017). Thus, exploring how personal resources and subjective dimensions may affect their work environment is a pivotal educational issue.

Creating a positive learning environment in schools has been a key target in the European agenda in recent years (European Union, 2013). Indeed, research indicates that positive experiences at school, linked to feelings of liking and being connected to the school context, serve as a shield against the risk of mental health problems (e.g., Patalay & Fitzsimons, 2018; Somersalo et al., 2002). This paper aligns well with the agenda goals, exploring teachers’ mentalized affectivity as a key personal resource in the psychological adjustment of a classroom (Bianco & Lecce, 2016; Valle et al., 2016; Nišponská, 2022; Valle et al., 2022). In addition, the roles served by two subjective dimensions of well-being (i.e., job and life satisfaction) and the teacher-level factors (teaching experience and school grade) were also investigated.

Preliminarily, the study examined the differences in the study’s variables between teachers in primary and lower secondary education. The results highlighted that teachers at primary schools reported a more positive classroom climate compared to their counterparts. This is in line with evidence (Wang & Dishion, 2012; Way et al., 2007) showing a problematic classroom climate in lower secondary schools due to the presence of both challenging social relationships and students’ perception of an unclear disciplinary structure (Malone et al., 2017). Nevertheless, the results are in contrast with the study by Somersalo et al. (2002) reporting a poor classroom climate at primary school due to the pupils’ emotional and behavioral problems that teachers must manage.

Correlational analyses supported the hypothesized direct and indirect paths. Among the teacher-related factors that may affect the classroom climate, only teaching experience and school grade were included as covariates in the parallel mediation model, and results supported their significant impact. Regarding teacher age, it was not included as a covariate due to its non-significant association with the study variables. Nevertheless, the decision is also consistent with current theoretical models of the classroom climate, which conceptualize its quality as a function of teachers’ personal resources and subjective dimensions (e.g., Pianta & Hamre, 2009), rather than of sociodemographic features such as age.

Our main results indicate the role, identified by the significant direct effect, played by the teachers’ mentalized affectivity in positively influencing the quality of the perceived classroom climate. Teachers’ mentalized affectivity is likely to affect the classroom climate, as those teachers who are competent in social and emotional domains tend to encourage cooperation, to be respectful of social exchanges, to try to promote prosocial acts among students, to build supportive relationships with their students, and to provide positive models to cope with conflict situations and misunderstandings (Jennings & Greenberg, 2009). Although preliminary and explorative in nature, the study’s results support the promotion of mentalized affectivity in teachers as a resource. It is worth noting that the majority of the studies (Valle et al., 2022; Byrne et al., 2020; Schwarzer et al., 2023; Chelouche-Dwek et al., 2026) trained teachers’ mentalizing ability with benefits; nevertheless, providing teachers with additional strategies for emotional regulation management could help them to better monitor their own emotions experienced in the school environment, not only helping to preserve students’ socio-emotional competencies (Bak et al., 2015; Bianco & Lecce, 2016) but also helping them to understand classroom dynamics and manage the classroom climate effectively (Buyse et al., 2009; Hamm et al., 2011).

Additionally, our work aimed to clarify some mechanisms through which teachers’ mentalized affectivity may impact the quality of the classroom climate. More precisely, we explored the role played by teachers’ job and life satisfaction as subjective dimensions of well-being. In this respect, our results on indirect paths is in line with previous research showing that mentalized affectivity is a protective factor for individuals’ subjective dimensions of well-being (Valle et al., 2016; Ballespí et al., 2019) and specifically for teachers (Safiye et al., 2023). Moreover, the results regarding the role of job and life satisfaction align well with the cluster of studies that focus on teachers’ well-being, adopting a complex perspective that aims to identify the reciprocal influences of personal, relational, and contextual factors that contribute to creating the classroom system (Nwoko et al., 2023; Dreer, 2023).

It is worth reflecting on the role played by the covariates. With regard to the role played by teacher-related factors, the length of teaching experience seems to be positively associated with the classroom climate, lending support to the idea that, beyond personal resources and subjective dimensions of well-being, experienced teachers acquire strategies aimed at improving the classroom climate and managing classroom dynamics (Karasova & Nehyba, 2025; Koth et al., 2008). An additional reflection is required on the non-significant role served by the school grade in the classroom climate. Although a difference in primary and lower secondary teachers’ perception of the classroom climate has been demonstrated (Malone et al., 2017; Collie et al., 2015; Cabello-Sanz et al., 2025), no significant effect of the teacher-level factor was reported. However, we must be cautious in interpreting this result. The interpersonal dynamics (student–student and teacher–student) during childhood and pre-adolescence may be affected by several factors that teachers must understand and manage. Thus, this path should be further investigated with research that could help to better understand the influence of the specific teacher-level factors investigated within the primary and lower secondary education contexts.

In summary, the present study confirms the importance of investing in teachers’ resources and well-being, given that they constitute both a vulnerable population and essential caregivers in the development of the younger generation. From an ecological perspective (Bronfenbrenner, 1979), it is not only the school system but also broader educational policy that must safeguard teachers’ well-being: although teachers invest their personal resources in their profession, they may nonetheless be undermined by broader political dynamics that lead them to disengage from their work and experience heightened fatigue in classroom management, particularly when working with children and pre-adolescents in crucial developmental stages.

Our findings therefore do not merely answer specific research questions, but also open avenues for further reflection and research aimed at identifying the features that the school environment must possess to enable teachers, and consequently their students, to fulfill their potential.

## 5. Study’s Strengths and Limitations

The novelty of the direct and indirect paths tested in the current study is a key strength of the study. To date, no studies have explored mentalized affectivity as a potential personal resource for primary and secondary teachers in improving the classroom climate. No studies considered the teacher’s job and life satisfaction as potential subjective dimensions of well-being that simultaneously mediate the direct path between mentalized affectivity and the classroom climate. In addition, we also explored teacher-level factors (teaching experience and school grade). Although devoting attention to female teachers limits the generalizability of the results, this sample restriction was in line with the national demographic data. It is also worth noting that focusing on this vulnerable population for mental health problems (Levante et al., 2025) is a pivotal study strength, informing possible gender-specific intervention programs. Indeed, the current study cannot determine whether the observed effects reflect a genuinely universal mechanism or one that is specific to, or amplified within, a female population. Therefore, the present findings should be interpreted as characterizing the mechanism among female teachers specifically, rather than among teachers in general. Lastly, the data collection was carried out during the COVID-19 pandemic, limiting not only the sample size due to the restrictions but also the results’ generalizability due to the manner of data collection during an extremely stressful period.

Although encouraging, the results should be interpreted with consideration to the study’s limitations.

Firstly, this study employed a cross-sectional design, and therefore future research should corroborate our main findings using a longitudinal design. Second, caution is also warranted when examining the measurement of the study’s variables through self-report. In the present study, the classroom climate was assessed through a task reflecting the perceptions of the teacher only, introducing a potential single-informant bias. In addition, only teachers’ perceptions of the classroom climate were considered. Future studies will also consider not only the students’ perspectives but also their outcomes in terms of socio-emotional and academic achievement within the Prosocial Classroom Model framework. The interaction between the teachers’ and the students’ perceptions of the classroom climate would be interesting to further investigate (Mitchell et al., 2010). Additionally, although the single-item assessments of job and life satisfaction have been previously applied successfully (De Carlo et al., 2024), the single items introduced a measurement error in the mediation model that future studies may overcome.

Third, a sample bias has to be considered among the study limitations due to the recruitment of female teachers only. Even if this sample composition aligns with the general situation in the Italian educational system (https://ec.europa.eu/eurostat/databrowser/view/educ_uoe_perd03/default/table?lang=en, accessed 20 November 2022), there may be different gender distributions in other countries. It is also worth noting that the sample size is limited to females, which may affect the generalizability of the results. Thus, they must be read with caution. In future research, this aspect should be taken into further account.

Finally, a set of confounding variables has not been assessed; that is, the number of students in the class, the type of teachers’ employment contracts (full-time versus part-time), or the number of work hours/per week. These may substantially affect the teachers’ satisfaction and, consequently, the classroom climate; thus, more investigations are required. Lastly, the power analysis computed for the present study used conventional values for effect size and desirable power. Considering the exploratory nature of the study, and since the literature does not report similar studies with reference to the expected effect size, conventional values lead to a conservative estimate of the sample size, ensuring that the general trend can be detected, even if it is not confirmatory (Lakens, 2013). Thus, acknowledging that the use of conventional values can introduce uncertainty if the real effect is small or if variability is high, the results should be interpreted as preliminary indications. Future studies may recalibrate the power using pilot data or results from future pilot studies.

## 6. Research and Educational Implications

Both research and educational fields may benefit from our results.

In the research field, our findings support the design of future research, which should extend the evaluation of the classroom climate to include students and incorporate measures that do not solely rely on teachers’ perceptions. Ideal candidates may be assessed based on the quality of social relations within the classroom, the psychological well-being of both teachers and students, and the attitudes of teachers and students toward learning. Moreover, in addressing the links between the mentalized affectivity of the teacher and the quality of the classroom climate, the current study did not explore all possible mechanisms of this effect beyond the teacher’s satisfaction, which could be considered in future studies. For example, the quality of relationships within the classroom, the educational challenges that teachers encounter daily at school (Jennings & Greenberg, 2009), and the inclusion of socio-emotional learning into the main objectives of lectures (Bak, 2012) could all be considered. Another intriguing possibility emerging from recent research is that the mentalizing abilities of the teacher may affect the mentalizing skills of the students (Bianco & Lecce, 2016; Lecce et al., 2022; Santelices et al., 2023; Valle et al., 2019, 2022), thereby positively influencing the relational climate of the classroom. All these potential mechanisms, through which mentalized affectivity may affect the quality of the classroom climate, should be explored in the future on heterogeneous samples to understand whether the mechanisms are sex-specific.

Given that the classroom climate appears to be especially beneficial for the psychological well-being of pupils with emotional and behavioral problems (Somersalo et al., 2002), future research should also investigate how students’ scholarly experiences can vary according to their teachers’ mentalized affectivity. For example, there is a possibility that the teacher’s mentalized affectivity could moderate the impact of students’ internalizing/externalizing problems, as already demonstrated in the child–parent relationship, both in typical and atypical development (Levante et al., 2023; Ensink et al., 2017; Bianco et al., 2021; Gray et al., 2017).

In the educational field, the results of the current study pave the way for enhancements in teachers’ mentalized affectivity, which could act as a protective factor against the potential detrimental effects described in the literature of poor mental health and competence in the social/emotional domain of teachers on classroom relationships, management, and climates (Jennings & Greenberg, 2009). Our work has potential applicative implications, suggesting that mentalized affectivity is an ability that deserves attention when devising educational and training paths for future teachers. For instance, targeted intervention studies on the promotion of mentalized affectivity may be designed. Effectiveness studies could evaluate training to support teachers in adopting a mentalizing position within their everyday relationships with pupils, encouraging them to consider what the pupil might be feeling rather than simply judging behavior; in addition, the effectiveness of training that supports teachers’ expression of their own mental states in the classroom may be useful to understand if this strategy might serve as an observable model of self-regulation for pupils. Indeed, mentalized affectivity is likely to guide how teachers take care of, interact with, and teach their students (Roeser et al., 2012). Therefore, it is essential to empower not only teachers’ mentalizing ability in general (Bak et al., 2015; Bak, 2012; Valle et al., 2016), but also, more specifically, mentalized affectivity, enhancing teachers’ awareness of their ability to convey mentalizing concepts and processes in the classroom (Bianco & Lecce, 2016; for a review on this issue, see Bianco & Castelli, 2023) and supporting students’ engagement.

## 7. Conclusions

Exploring the factors that affect the classroom climate is an essential educational issue. The current study emphasized mentalized affectivity as a potential protective factor for teachers in improving the classroom climate, which, indirectly, may enhance the teaching approach and students’ engagement. In addition, job and life satisfaction played a mediating role in the hypothesized relationship, supporting the need to design intervention programs that promote not only personal resources but also individual attitudes. The results achieved in the current study suggest that mentalized affectivity may be a reliable individual resource in improving personal and professional well-being, which, in turn, may have positive cascade effects on students’ academic outcomes and well-being.

Taken together, these findings offer a novel contribution to the existing literature by identifying job and life satisfaction as potential subjective dimensions of teachers’ well-being that influence the classroom climate. While mentalized affectivity has been extensively studied in relation to individual emotional well-being and the classroom climate has been separately linked to teachers’ job and life satisfaction, the present study is, to the best of our knowledge, among the first to integrate these two lines of research empirically, showing that teachers’ capacity to identify, process, and express their own emotions extends beyond individual functioning to shape the relational quality of the classroom environment through the satisfaction teachers derive from their work and their lives more broadly.

## Figures and Tables

**Table 1:** Results of group comparison between psychological dimensions.

	Primary School Teachers M (SD)	Lower Secondary Education Teachers M (SD)	*U*-Test; *p*-Value
**Mentalized Affectivity**	4.79 (0.61)	4.75 (0.60)	*U* = 15.272; *p* = 0.410
**Classroom Climate**	4.68 (0.60)	4.36 (0.55)	*U* = 11.526; *p* < 0.001
**Job Satisfaction**	7.91 (1.17)	7.67 (1.49)	*U* = 15.393; *p* = 0.463
**Life Satisfaction**	8.02 (1.47)	7.99 (1.39)	*U* = 15.055; *p* = 0.282

**Table 2:** Correlation analysis between the study’s variables.

	Length of Teaching Experience	(1)	(2)	(3)	(4)	(5)
**Length of teaching experience**	-	0.743 ***	−0.314 ***	0.377 ***	−0.158 *	−0.265 ***
**Teachers’ age (1)**		-	−0.094	0.129	−0.045	−0.098
**Mentalized affectivity (2)**	−0.147	0.119	-	0.539 ***	0.210 **	0.454 ***
**Classroom climate (3)**	0.238 **	0.193	0.237 **	-	0.026 ***	0.421 ***
**Job satisfaction (4)**	−0.048	0.007	0.439 ***	0.238 ***	-	0.433 ***
**Life satisfaction (5)**	−0.027	0.108	0.477 ***	0.025 ***	0.738 **	-

Note: * *p* < 0.05; ** *p* < 0.01; *** *p* < 0.001. Above the diagonal, the correlations for the subsample of teachers in primary schools are reported. In contrast, below the diagonal, the correlations for the subsample of teachers in lower secondary education are reported.

**Table 3:** Standardized coefficients of indirect effects.

			95% Bootstrap CI	
	Beta	BootSE	BootLLCI	BootULCI
**Total effect**	0.060	0.007	0.047	0.074
**Direct path**				
Mentalized affectivity → classroom climate	0.050	0.007	0.036	0.065
**Indirect path**				
Mentalized affectivity → job satisfaction → classroom climate	0.041	0.018	0.007	0.076
Mentalized affectivity → life satisfaction → classroom climate	0.093	0.042	0.001	0.165
Mentalized affectivity → job satisfaction → life satisfaction → classroom climate	0.018	0.011	0.001	0.044

## Data Availability

Data will be shared upon request.
